# 3D Nanowire Pt Catalysts with Enhanced Stability for the Oxygen Reduction Reaction

**DOI:** 10.1021/acsomega.4c06385

**Published:** 2025-02-21

**Authors:** Joshua S. White, Wanli Liu, Samuel C. Perry, Samina Akbar, Diego Alba-Venero, Nicholas J. Terrill, Adam Squires, Iris Nandhakumar

**Affiliations:** † Department of Chemistry, University of Southampton, Southampton SO17 1BJ, U.K.; ‡ Department of Chemistry, University of Bath, South Building, Soldier Down Ln, Claverton Down, Bath BA2 7AY, U.K.; § Rutherford Appleton Laboratory, ISIS Neutron and Muon Source, Didcot OX11 0QX, U.K.; ∥ Diamond Light Source, Diamond House, Harwell Science and Innovation Campus, Didcot, Oxfordshire OX11 0DE, U.K.

## Abstract

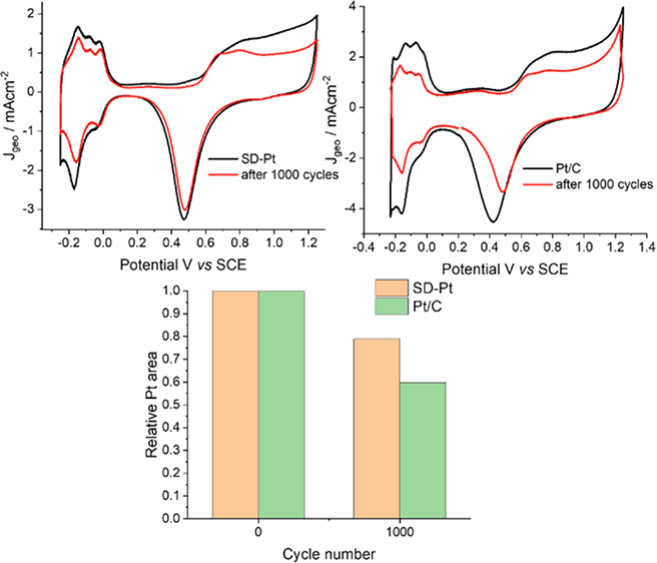

We report that self-supporting mesoporous platinum 3D nanowires with a single diamond (SD) morphology and a high specific surface area of 40.4 m^2^ g^–1^ demonstrated enhanced stability toward the oxygen reduction reaction (ORR). These were found to be superior to commercially available carbon-supported Pt nanoparticles (Pt/C). After 1000 potential cycles, there was a 21% loss in surface area for SD-Pt, as compared with a 40.3% loss for Pt/C with no reduction in their half-wave potential (measured at *J* = 3.0 mA cm^–2^), whereas the Pt/C catalyst showed a 11.9 mV decrease. Our findings revealed that our SD-Pt thin films also exhibited excellent ORR activity, which offers significant potential for their application as high-performance cathode materials in alkaline fuel cells.

## Introduction

Fuel cells are pivotal to the future of sustainable energy production as they can harness fuels derived from renewable sources with the potential to generate energy with minimal greenhouse gas emissions, achieving near zero environmental impact.^1^ The oxygen reduction reaction (ORR) plays a crucial role in electrochemistry for energy conversion in fuel cells. However, its sluggish kinetics at the cathode is a major limitation of their application. As a result, there is a clear need for developing economically viable and highly effective ORR electrocatalysts that can drive the four-electron transfer ORR.

The power output in fuel cells and their durability is dependent on the cathode performance as this is where most of the polarization losses take place.^2^ The ORR can occur via two different pathways: a direct 4-electron pathway (eq 1) or an indirect pathway given by eqs 2 and 3.
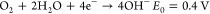
1
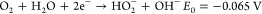
2
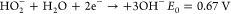
3

Priority is given to developing catalysts that promote the more efficient four-electron direct pathway. The performance of electrocatalytic ORR catalysts is primarily assessed by using the onset potential, half-wave potential, and diffusion-limiting current characteristics. In general, specific/mass activity at relatively higher potentials is employed to evaluate the efficiency of the ORR catalysts. Current literature appears to be mainly focusing on investigating Pt nanoparticles supported on a carbon black matrix (Pt/C).^3,4^ These catalysts display a high activity for the ORR, which proceeds via the direct four-electron transfer pathway at an onset potential of 0.94 V_RHE_ and a kinetic current density of 31.5 mA cm^–2^ at 0.8 Vs RHE.^5^

While platinum (Pt) is an excellent electrocatalyst, its high cost and limited availability make it imperative to reduce the amount being used.^4,6^ One of the most prominent strategies is to increase the catalyst surface area, which lowers Pt loading and also enhances its performance. This can be achieved by increasing the surface-to-volume ratio by controlling the morphology in micro-/nanostructures.^7^ Improvement in the specific activity has been reported for mesostructured Pt films.^8^ Yao et al. reported an unusual size effect in Pt nanowires (NWs) with adjustable diameters, where both activity and durability for the ORR increased consistently as the diameter decreased from 2.4 to 1.1 nm.^9^ Reducing the amount of Pt while improving its catalytic activity is another strategy adopted in recent years. Efforts are being made to optimize the ORR performance by carefully controlling the Pt particle size. Changing the size and morphology may lead to exposure of the desired crystal facets. Altering the size and morphology of the catalyst can enhance both the exposure of its active sites and its overall activity. It has been reported that PtNi (111) exhibits catalytic activity, which is 90 times greater than that of commercial Pt/C.^10^

To create highly efficient Pt-based catalysts, it is essential to optimize their activity and stability. However, there is multiple evidence from the literature that current materials exhibit low stability, as evidenced by performance deterioration after galvanostatic experiments and performance fluctuations following voltage cycling. The instability of Pt/C catalysts with respect to the ORR has been attributed to a loss in electrochemical surface area (ECSA),^11^ particle migration and agglomeration,^12^ the corrosion of the carbon support (leading to a loss of Pt nanoparticles),^13^ and dissolution of Pt into the electrolyte.^14^ To improve the durability of Pt/C ORR catalysts, alloys of Pt with other transition metals have been produced,^15^ or core–shell nanoparticles^16^ have been produced using metal oxide supports rather than carbon.^17^ Alternatively, unsupported Pt catalysts have also been prepared,^8,18,19^ which avoid corrosion of the carbon support.^18^

**Figure 1:**
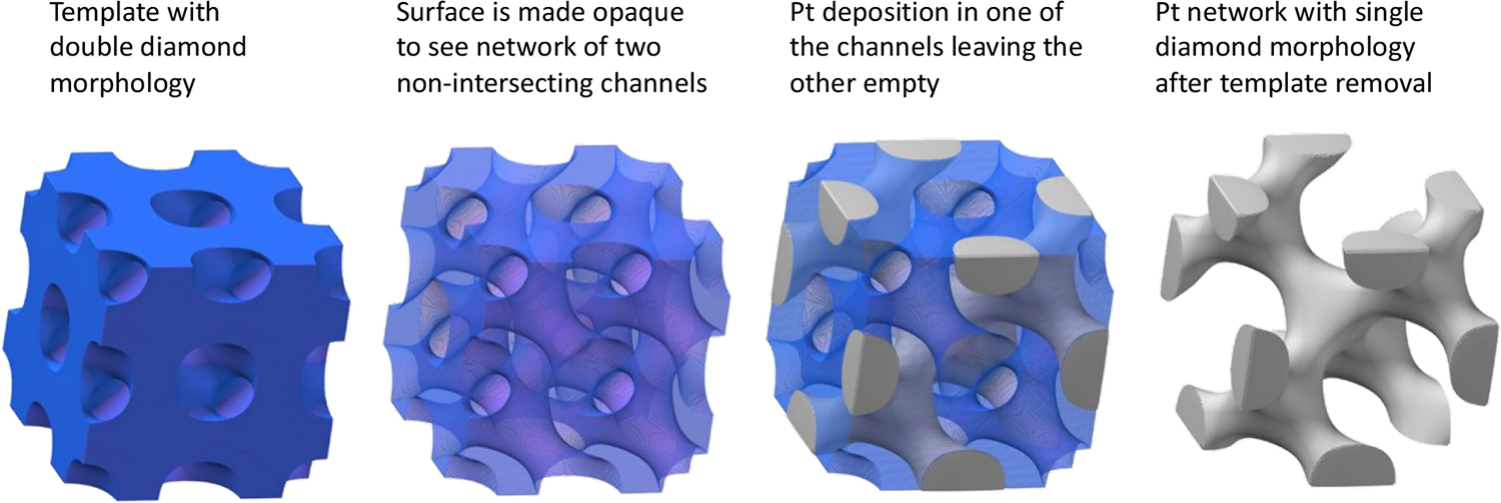
Electrodeposition of single diamond Pt (SD-Pt) within the phytantriol template.

In this study, we report the fabrication of 3D ordered mesoporous Pt with a single diamond (SD) morphology (cf. Figure 1) via soft-templated electrodeposition that resulted in a much-improved stability for the ORR compared to Pt/C. In this material, both the external and internal surfaces are accessible, resulting in an inherently high active surface area, which eliminates the need for the carbon support. Furthermore, as this is composed of a 3D continuous network of interconnected nanowires, it is much more corrosion-resistant and can maintain electrical contact. Further, this fabrication method provides a green “one-pot” synthesis method for producing a catalyst that exhibits competitive catalytic performance toward the ORR and enhanced stability over state-of-the-art commercial Pt/C.

## Results and Discussion

**Figure 2:**
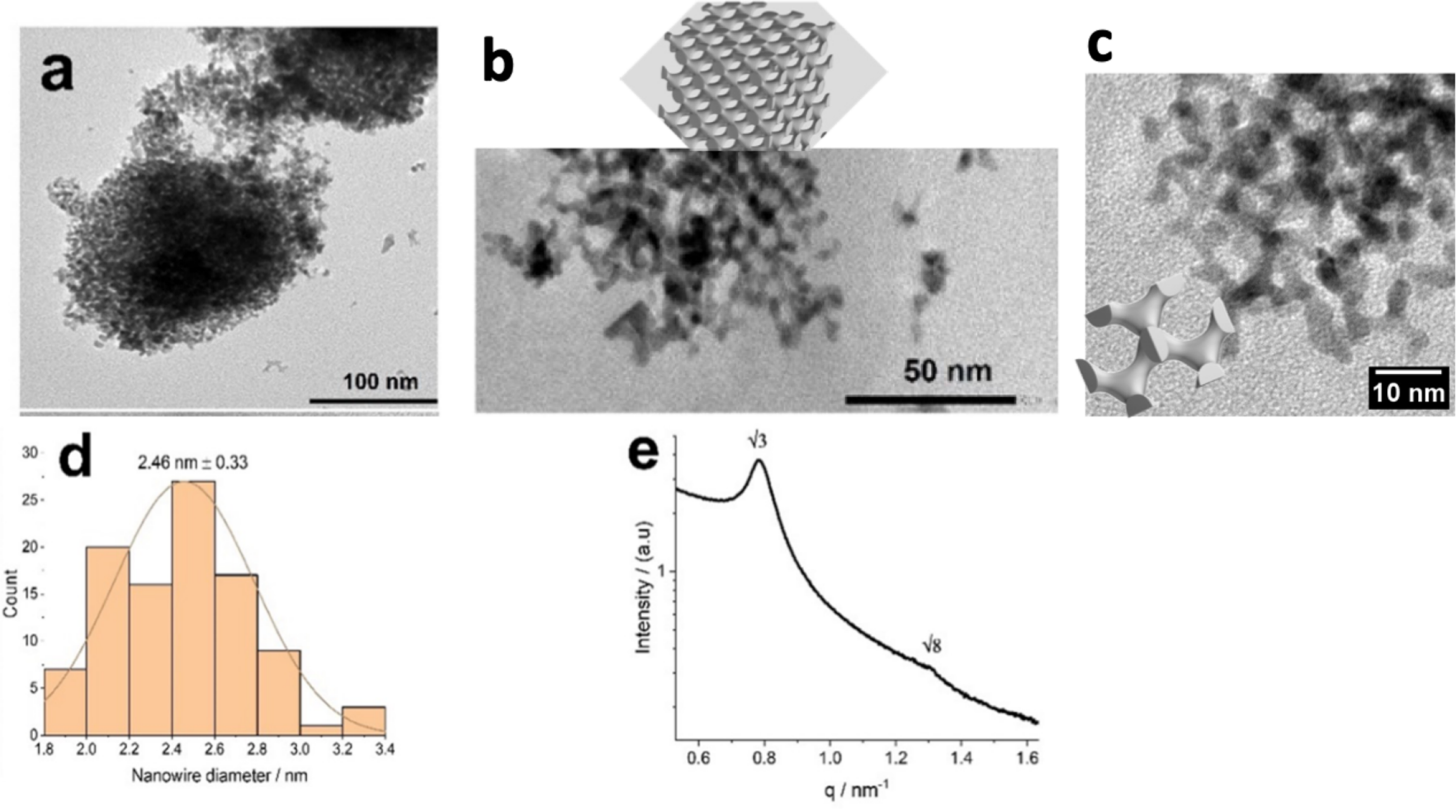
(a) 1D-integrated SAXS pattern of the SD-Pt thin film electrodeposited through a phytantriol-modified Au-DVD electrode after template removal. (b,c) TEM images at different magnifications of SD-Pt with superimposed matching simulations drawn to scale along with nanowire diameter distribution (d,e).

Transmission electron microscopy (TEM) images (Figures 2a–c and S1) confirmed that the electrodeposited Pt is stable after template removal and forms a mesoporous interconnected nanowire network. Small-angle X-ray scattering (SAXS) (Figure 2e; collected on I22, the beamline at Diamond Light source, experiment number NT33748)^20^ confirmed the SD morphology of the deposited Pt.

The results from SAXS were used to obtain structural information on SD-Pt, which is summarized in Table 1.

**Table 1:** Lattice Parameter, Nanowire Diameter, and Pore Width of Single Diamond Pt Measured from TEM and SAXS

lattice parameter (SAXS) [nm^–1^]	estimated channel diameter (SAXS) [nm]	estimated pore size [nm]	nanowire diameter (TEM) [nm]
13.6 ± 0.2	2.8 ± 0.2	6.8 ± 0.2	2.46 ± 0.3

The lattice parameter of SD-Pt was found to be 13.2 nm, which corresponds to asymmetric deposition through a single channel network of a double diamond structure with a lattice parameter *a* = 6.9 ± 0.1 nm (a 1D SAXS pattern is shown in the Supporting Information, Figure S2). The water channel diameter *d*_w_ in the double diamond phase is calculated using eq 4.^21^
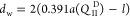
4

Assuming a lipid monolayer thickness (*l*) of 1.36 nm for phytantriol,^22^*d*_w_ was calculated to be 2.8 nm ±0.1. TEM measurements indicated an average nanowire diameter of 2.5 nm, which is in good agreement with the calculated value, indicating that the size of the water channels within the diamond template is transferred to the deposited nanowires (cf. Figure 2d). The pore size was determined by calculating , resulting in a value of 6.8 nm. These values are in excellent agreement with previous literature values.^23^

**Figure 3:**
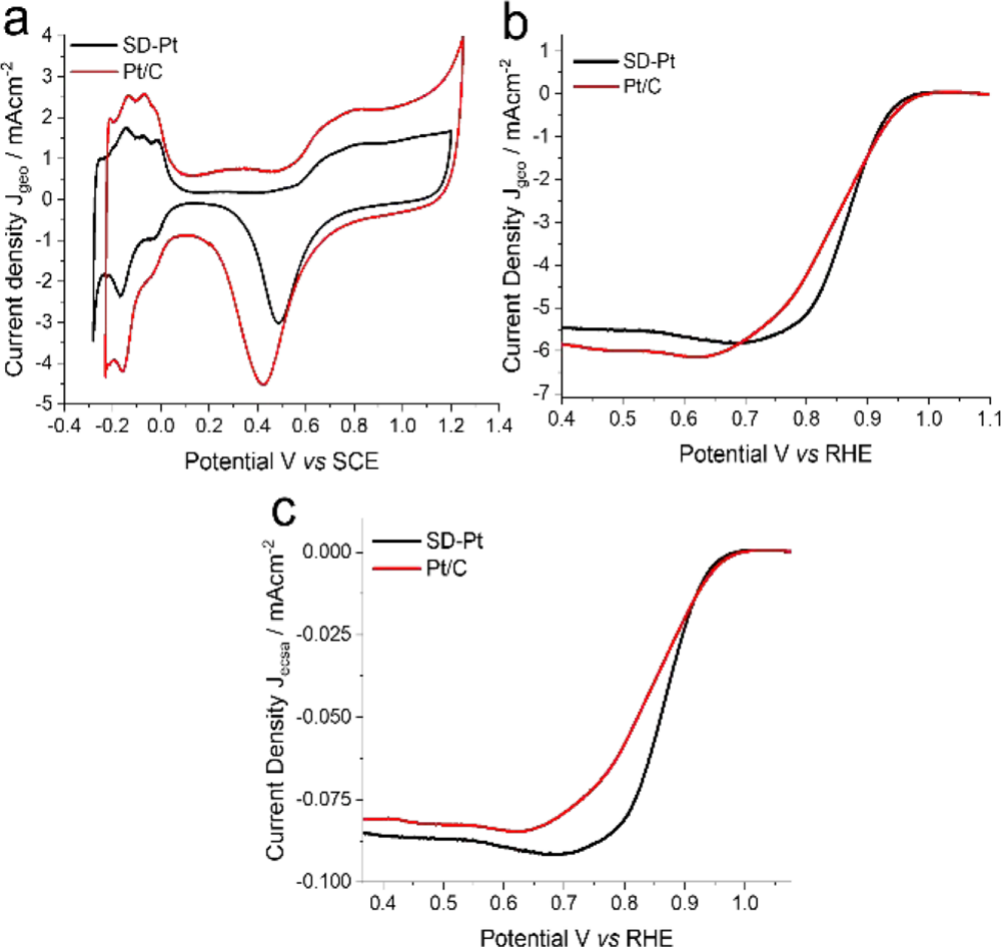
(a) Cyclic voltammograms of SD-Pt and Pt/C taken in 0.5 M H_2_SO_4_ at room temperature at a scan rate of 20 mV s^–1^. Linear sweep voltammograms of SD-Pt and Pt/C were taken in 0.1 M KOH at room temperature at a scan rate of 10 mV s^–1^ and a rotation speed of 1600 rpm, normalized by geometric surface area (b) and electroactive surface area (c).

The electrochemical properties of the as-prepared SD-Pt and Pt/C catalysts were measured for comparison. Cyclic voltammetry studies performed in 0.5 M H_2_SO_4_ at a sweep rate of 20 mV s^–1^ were used to determine the electrochemically active surface area and specific surface area, as shown in Figure 3a. The specific surface area of SD-Pt was calculated to be 40.4 m^2^ g^–1^, which is comparable to that measured for Pt/C at 46.2 m^2^ g^–1^.

The catalytic activity of SD-Pt for the ORR was assessed by using an Autolab rotating disc electrode (RDE) 2 in O_2_-saturated 0.1 M KOH. Cycling voltammetry was performed at a scan rate of 10 mV s^–1^ at a rotation rate of 1600 rpm on both SD-Pt and Pt/C.

It can be seen that for both materials, the onset potential for the ORR is very similar at 1.0 and 0.98 V vs RHE for Pt/C and SD-Pt, respectively. It can also be observed that there is a positive shift in the half-wave potential (*E*_1/2_) measured at *J* = 3.0 mA cm^–2^ of 20.7 mV for SD-Pt compared to Pt/C. This positive shift is in agreement with the literature on double gyroid Pt.^8^ The Pt/C exhibits a limiting current density higher than that of SD-Pt initially. However, upon normalizing the current using the real surface area of the films (determined from hydrogen underpotential deposition), it becomes evident that the limiting current density is higher for SD-Pt. These results indicate that there is an improved activity for SD-Pt in the ORR. Kibsgaard et al. reported that the improved per site activity for self-supported mesoporous Pt films most likely originates from a smaller fraction of undercoordinated surface sites compared to Pt/C.^8^Table 2 compares the performance of SD-Pt against recent advanced catalysts reported in the literature. It is apparent that SD-Pt has similar performance regarding the onset potential and half-wave potential when compared with other catalysts.

**Table 2:** Comparative Analysis of the Performance of Recent Catalysts for the Oxygen Reduction Reaction (ORR)

catalyst	electrolyte	*E*_onset_ (V vs RHE)	*E*_1/2_ (V vs RHE)	reference
SD-Pt	0.1 M KOH	0.98	0.87	this work
Pt/C	0.1 M KOH	1.00	0.84	this work
Pt_2_Pd_1_	0.1 M KOH	0.97	0.89	(24)
Fe_3_Ptt/N@C	0.1 M KOH	0.98	0.88	(25)
Pt_37_Cu_56_Au_7_	0.1 M KOH		0.91	(26)
Pd_3_Au/C	0.1 M KOH	0.99	0.87	(27)
SA-PtCoF	1 M KOH	0.95	0.88	(28)
IrMn/Fe3Mo3C	0.1 M KOH	1.03	0.89	(29)
CoSMe-0.5–800	0.1 M NaOH	0.95	0.85	(30)
CoS NWs@NSC-2	0.1 M KOH	0.93	0.84	(31)
ZrN	0.1 M KOH	0.93	0.80	(32)
NDC1000	0.1 M KOH	0.96	0.86	(33)
NC-Co SA	1 M KOH	1.00	0.87	(34)

**Figure 4:**
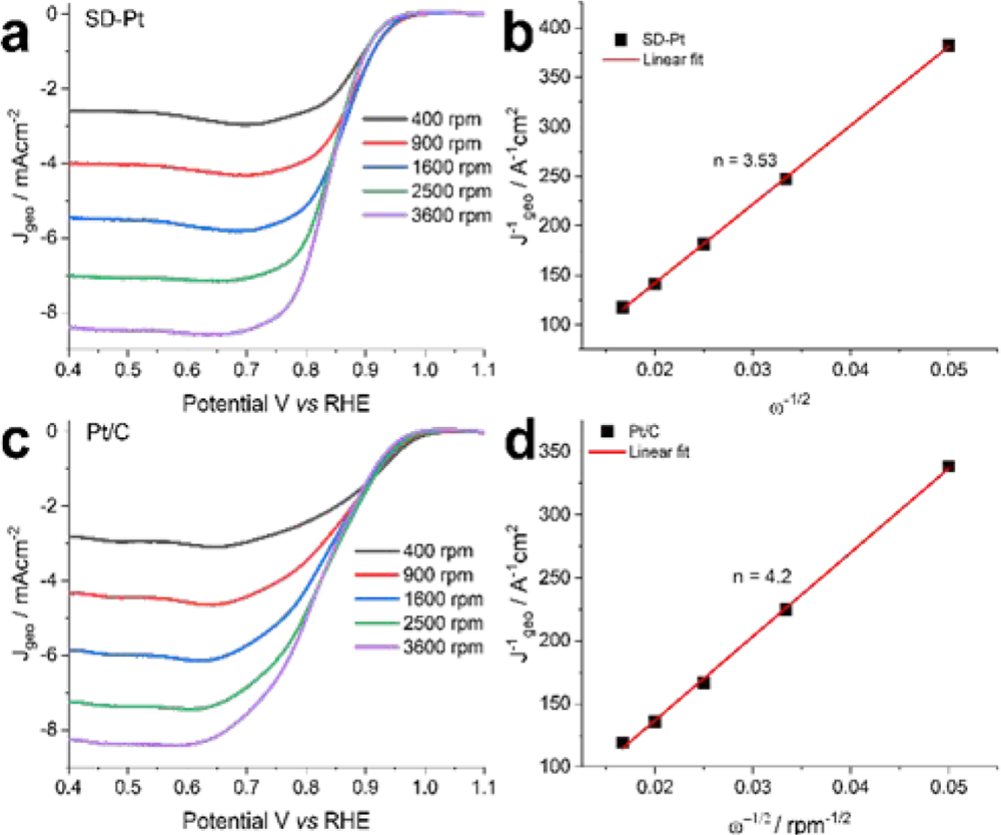
LSV measurements taken at different rotation rates in O_2_-saturated 0.1 M KOH at a scan rate of 10 mV s^–1^ along with the associated Koutecky–Levich plot of SD-Pt (a,b) and Pt/C (c,d).

A Koutecky–Levich analysis was performed to determine the ORR pathway under alkaline conditions from RDE measurements obtained at rotating speeds from 400 to 3600 rpm at a scan rate of 10 mV s^–1^ (Figure 4a,c).

The electron transfer number (*n*) was then determined from the slope of the Koutecky–Levich plots in combination with eqs 5 and 6:
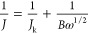
5
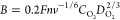
6where *J* is the measured current density, *J*_k_ is the kinetic current density, ω is the rotation speed in rpm, *B* is the reciprocal of the slope, *n* is the number of electrons transferred, *F* is the Faraday constant (96485 C mol^–1^), *D*_O2_ is the diffusion coefficient of O_2_ in 0.1 M KOH (1.98 × 10^–5^ cm^2^ s^–1^), *v* is the kinetic viscosity (0.01 cm^2^ s^–1^), and *C*_O2_ is the concentration of O_2_ in 0.1 M KOH (1.48 × 10^3^ mol L^–1^). The calculated number of electrons transferred per oxygen molecule was 3.5 and 4.2 for SD-Pt and Pt/C, respectively. Since a three-electron pathway is physically impossible, the ORR is likely occurring through the 4-electron pathway, considering possible measurement errors. This is consistent with literature reports on the ORR of Pt in alkaline media.^35^ In a study reported earlier, for values less than 3.5 (calculated for bimetallic Pt–Ni nanomaterials), a mixed two- and four-electron transfer was suggested.^36^

**Figure 5:**
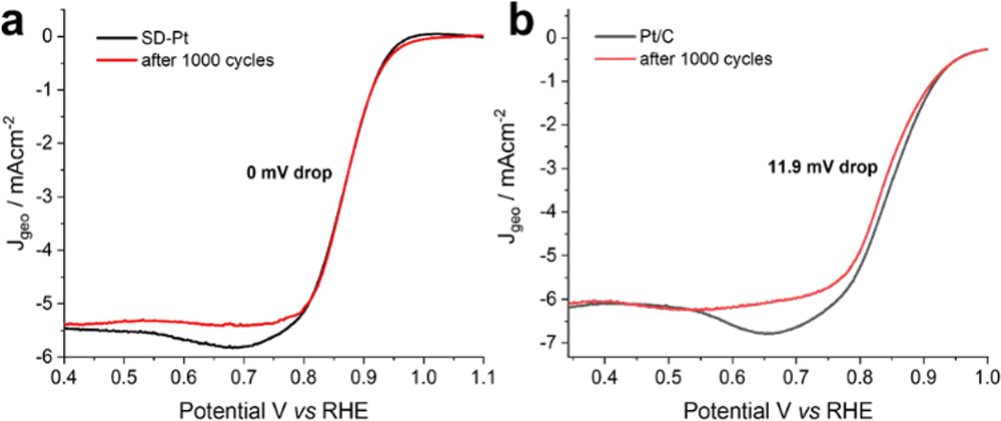
LSV curves of SD-Pt (a) and Pt/C (b) in O_2_-saturated 0.1 M KOH at a scan rate of 10 mV s^–1^ and 1600 rpm before and after potential cycling for 1000 cycles.

As previously mentioned, one major issue facing the development of fuel cells is catalyst stability. To investigate the stability of SD-Pt, durability tests in the form of potential cycling were undertaken. The potential was cycled between 0.1 and −0.5 V vs SCE at a scan rate of 2 V s^–1^ in O_2_-saturated KOH at room temperature. LSV was carried out in fresh O_2_-saturated 0.1 M KOH immediately after 1000 cycles in N_2_-saturated 0.5 M H_2_SO_4_. The LSV curves in Figure 5 show that after 1000 cycles, there was no drop in the half-wave potential (measured at *J* = 3.0 mA cm^–2^) for SD-Pt compared to a drop of 11.9 mV for the Pt/C catalyst. These results indicate that over 1000 cycles, SD-Pt has significantly better durability for the ORR compared to the commercial catalyst.

**Figure 6:**
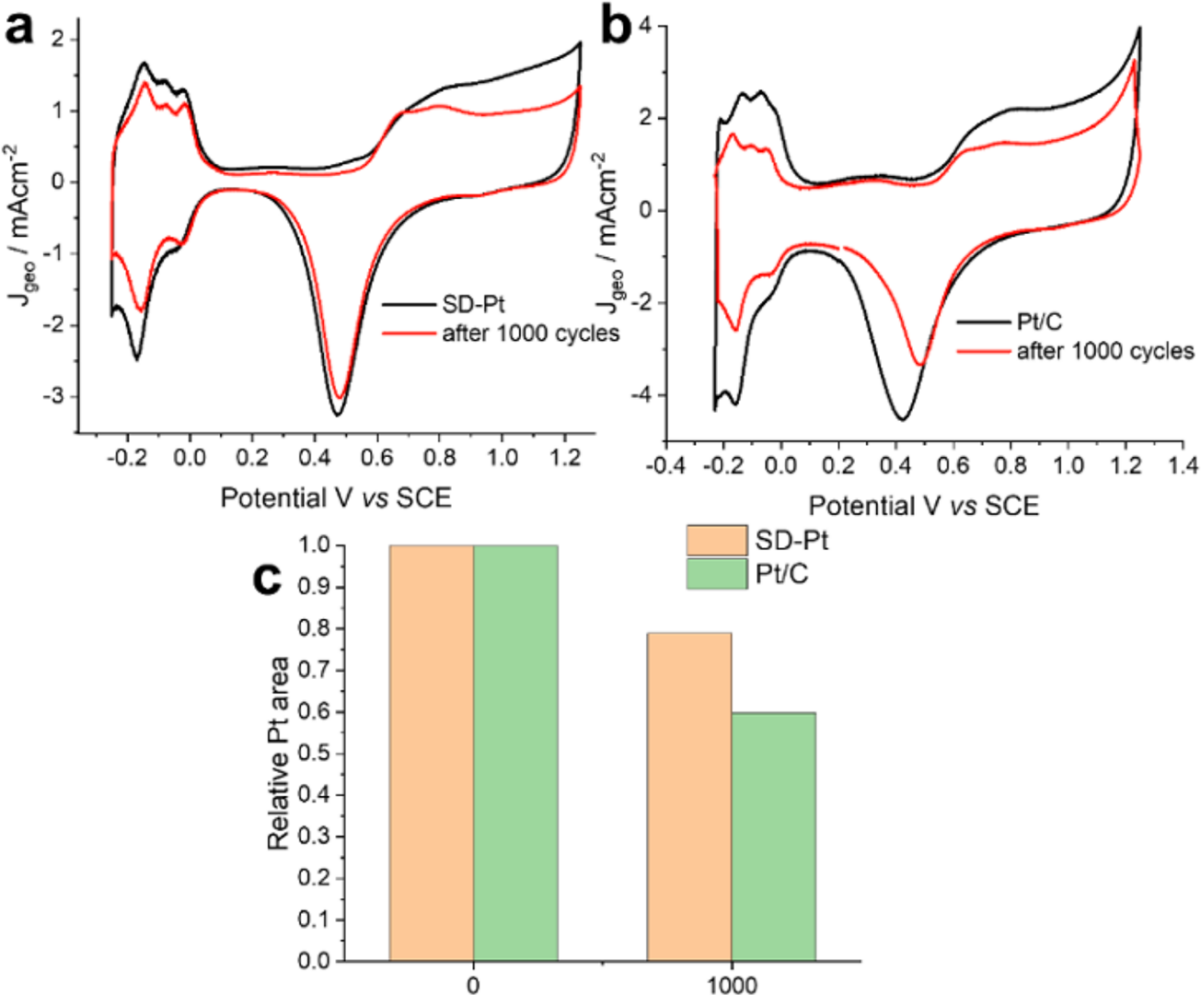
Comparison of the electroactive surface area degradation of SD-Pt (a) and Pt/C (b) with potential cycling taken by cyclic voltammetry in N_2_-saturated 0.5 M H_2_SO_4_ at 50 mV s^–1^. Relative loss in Pt surface area with the number of cycles measured by hydrogen underpotential deposition (c).

Figure 6 shows the relative loss in the electroactive surface area of SD-Pt in comparison to that of Pt/C before and after potential cycling. After 1000 cycles, there is a significant drop in surface area for Pt/C of around 40%, whereas SD-Pt only has a loss of 20%, implying better durability.

From these stability tests, we can conclude that SD-Pt offers a better overall performance for the ORR due to its higher durability and reduced loss in electroactive surface area after 1000 potential cycles compared to commercial Pt/C. In addition, the SD-Pt catalyst can be easily and rapidly prepared through a one-pot synthesis method compared to alternative methodologies for producing mesoporous Pt from hard templates and commercial Pt/C, which are not as environmentally friendly.

## Conclusions

In conclusion, our study has demonstrated that a self-supported mesoporous Pt catalyst with a single diamond architecture exhibited excellent stability toward the ORR superior to that of commercial Pt/C while simultaneously displaying high catalytic performance. These findings suggest that this material can potentially be incorporated as a promising catalyst material in high-performance fuel cells in the future.

## Materials and Methods

All of the compounds were used as received. Phytantriol was purchased from TCI Europe (98% purity), while hexachloroplatinic acid (HCPA) solution (8 wt % in water) was purchased from Aldrich. 0.5 M sulfuric acid was prepared by dilution from Merck p.a. grade concentrated acid. 0.1 M potassium hydroxide was prepared by dissolution of KOH purchased from Merck. All solutions were prepared in ultrapure Milli-Q water. Electrochemical studies were carried out in a standard three-electrode cell composed of a platinum gauze counter electrode, an SCE reference electrode, and a 3 mm Au disc rotating disc electrode.

Single diamond Pt was synthesized following the procedure by Akbar et al.^23^ Au disc electrodes (diameter = 3 mm) are used for electrochemical investigations. For SAXS and TEM analysis, gold archival DVDs of area ∼1 cm^2^ from Delkin Devices are used as working electrodes. Working electrodes were coated with a thin layer of phytantriol by dipping into an ethanolic mixture phytantriol (w/w ratio of 1:2), followed by drying under ambient conditions. Phytantriol-coated electrodes were soaked in an HCPA solution for not less than 10 min prior to deposition. For deposition, the potential was stepped from +0.6 to −0.246 V vs the SCE reference electrode in excess HCPA solution. After deposition, the phytantriol layer was washed away by rinsing with ethanol and water.

In order to prepare the Pt/C benchmark catalyst, a measured amount of 20 wt % Pt on Vulcan carbon (XC72R) (purchased from Johnson Matthey) was measured out and mixed with 0.9 mL of DI water, 0.5 mL of IPA, and 0.5 mL of Nafion (used as purchased from Aldrich) to create an ink. The ink was then placed on a 3 mm Au RDE tip and allowed to dry to create the Pt/C catalyst. For physical characterization, SD-Pt was electrodeposited onto electrodes cut for use from archival gold DVDs from Delkin Devices.

SAXS experiments were performed at Diamond Light Source using beamline I22 in transmission mode with a detector distance of 4752 mm and an energy of 12.4 keV. TEM analysis was carried out using the model JSM-2100PLUS.

## Supporting Information Available

TEM images of SD-platinum and 1D-integrated SAXS pattern of phytantriol (PDF)
