# Supplementary material for: 3D Nanowire Pt Catalysts with Enhanced Stability for the Oxygen Reduction Reaction
Source: ACS Omega. 2025 Feb 21;10(8):8082–8. doi: 10.1021/acsomega.4c06385 (PMC11886918; doi:10.1021/acsomega.4c06385)
Supplement: Supplementary file 1 — ao4c06385_si_001.pdf [file ao4c06385_si_001.pdf]

3D nanowire Pt catalysts with enhanced stability for the oxygen reduction reaction

Joshua S. White,[a] Wanli Liu,[b] Samuel C. Perry,[a] Samina Akbar,[a] Diego Alba-Venero,[c] Nicholas J. Terrill,[d] Adam Squires,[b] Iris Nandhakumar[a]*

[a]Department of Chemistry, University of Southampton, Southampton, SO17 1BJ. UK

[a]Department of Chemistry, University of Bath, South Building, Soldier Down Ln, Claverton Down, Bath, UK

[c]ISIS Neutron and Muon Source, Rutherford Appleton Laboratory, Didcot OX11 0QX, U.K

[d]Diamond Light Source, Diamond House, Harwell Science and Innovation Campus, Didcot, Oxfordshire OX11 0DE, U.K.

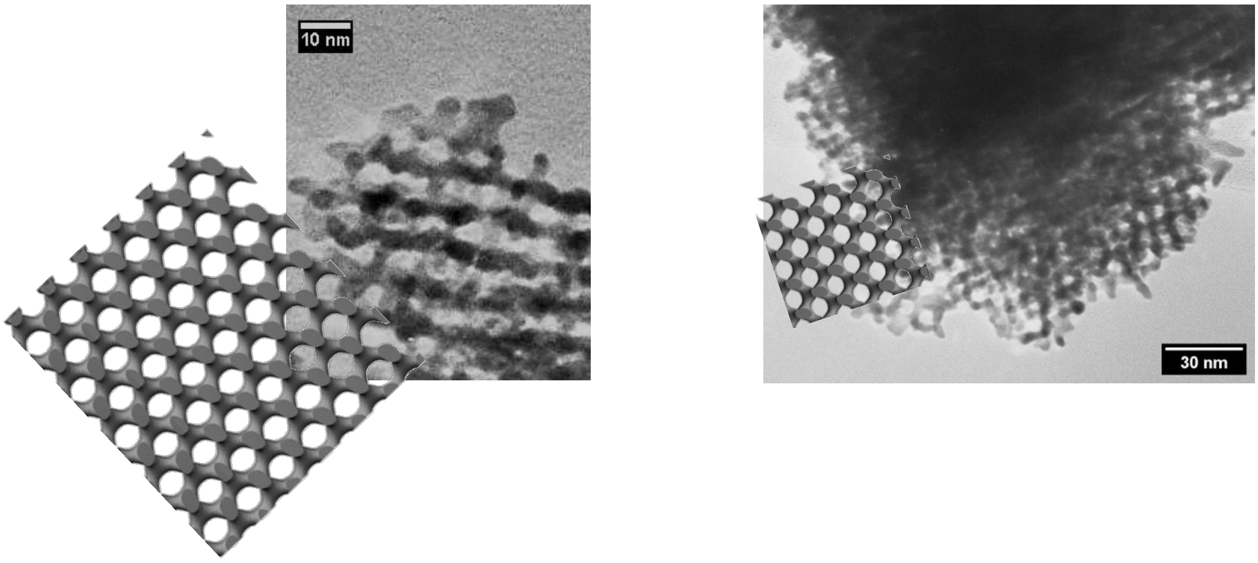

Figure S1:TEM images of SD-Platinum with matching simulations.

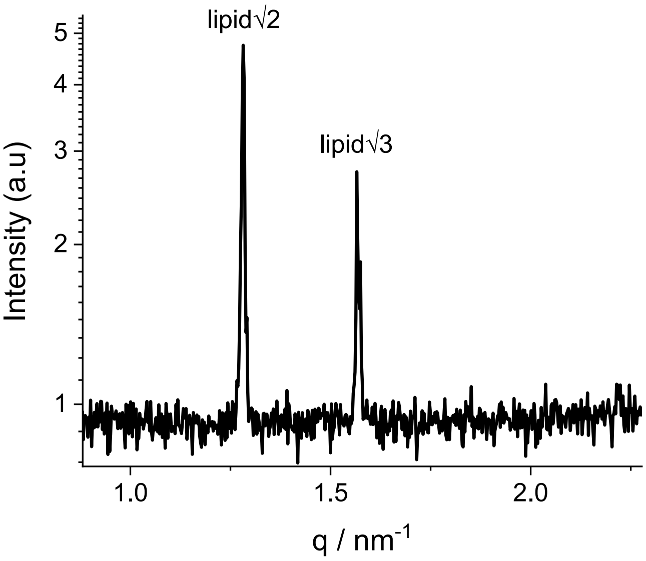

Figure S2: 1D integrated SAXS pattern of phytantriol in excess HCPA solution collected on the I22 beamline at Diamond Light source (experiment number NT33748). Lattice parameter value is estimated to be 6.9±0.1 nm.
